# Diagnostic accuracy of cyst fluid amphiregulin in pancreatic cysts

**DOI:** 10.1186/1471-230X-12-15

**Published:** 2012-02-14

**Authors:** May T Tun, Reetesh K Pai, Shirley Kwok, Aiwen Dong, Aparna Gupta, Brendan C Visser, Jeff A Norton, George A Poultsides, Subhas Banerjee, Jacques Van Dam, Ann M Chen, Shai Friedland, Brennan A Scott, Rahul Verma, Anson W Lowe, Walter G Park

**Affiliations:** 1 Department of Medicine, Stanford University, Stanford, CA 94305, USA; 2 Department of Pathology, Stanford University, Stanford, CA 94305, USA; 3 Department of General Surgery, Stanford University, Stanford, CA 94305, USA; 4 Palo Alto Medical Foundation, Palo Alto, CA 94301, USA; 5 Department of Medicine, University of Southern California, Los Angeles, CA 90033, USA; 6 Always Building, Room M211, 300 Pasteur Drive, Stanford, CA 94305-5187, USA

## Abstract

**Background:**

Accurate tests to diagnose adenocarcinoma and high-grade dysplasia among mucinous pancreatic cysts are clinically needed. This study evaluated the diagnostic utility of amphiregulin (AREG) as a pancreatic cyst fluid biomarker to differentiate non-mucinous, benign mucinous, and malignant mucinous cysts.

**Methods:**

A single-center retrospective study to evaluate AREG levels in pancreatic cyst fluid by ELISA from 33 patients with a histological gold standard was performed.

**Results:**

Among the cyst fluid samples, the median (IQR) AREG levels for non-mucinous (n = 6), benign mucinous (n = 15), and cancerous cysts (n = 15) were 85 pg/ml (47-168), 63 pg/ml (30-847), and 986 pg/ml (417-3160), respectively. A significant difference between benign mucinous and malignant mucinous cysts was observed (*p *= 0.025). AREG levels greater than 300 pg/ml possessed a diagnostic accuracy for cancer or high-grade dysplasia of 78% (sensitivity 83%, specificity 73%).

**Conclusion:**

Cyst fluid AREG levels are significantly higher in cancerous and high-grade dysplastic cysts compared to benign mucinous cysts. Thus AREG exhibits potential clinical utility in the evaluation of pancreatic cysts.

## Background

Pancreatic cysts are increasingly recognized from routine use of computed tomography and magnetic resonance imaging with current prevalence estimates of 2% in the population, rising to approximately 8% in the elderly [1,2]. Appropriate diagnosis and management of these cysts is clinically important because approximately half may have potential for malignant transformation to pancreatic adenocarcinoma - a cancer associated with an overall 5-year survival rate of 5% [3,4]. Cysts with malignant potential include mucinous cystic neoplasms (MCN) and intraductal papillary mucinous neoplasms (IPMN).

Various diagnostic tests, including endoscopic ultrasound (EUS), are employed to facilitate diagnosis and management of pancreatic cysts [5,6]. EUS guided aspiration of cyst fluid provides an opportunity to evaluate for tumor markers such as carcinoembryonic antigen (CEA) that can differentiate mucinous from non-mucinous cysts with reasonable accuracy. CEA cannot, however, accurately differentiate pre-malignant cysts from malignant cysts [7]. Further, cyst fluid cytology also possesses low sensitivity for diagnosing malignancy [8]. Because progression to cancer may be slow and variable among pre-malignant mucinous cysts, a biomarker that identifies cysts with cancer or high-grade dysplasia may have clinical value by identifying which patients may benefit from immediate consideration for surgery [9-12].

In this study, the diagnostic utility of the secreted epidermal growth factor receptor ligand, amphiregulin (AREG), was explored as a cyst fluid biomarker for the presence of malignancy in pancreatic cysts. AREG was chosen based on previous gene expression studies that identified enhanced Anterior Gradient 2 (*AGR2*) expression in all pancreatic adenocarcinomas [13]. *AGR2 *stimulates adenocarcinoma cell growth and supports the development of many features associated with malignant transformation [14,15]. A recent study demonstrated that *AGR2*'s growth promoting properties are achieved through its induction of *AREG *expression in adenocarcinoma cells [16]. As a secreted molecule, we hypothesized that the AREG concentration within the cyst fluid of adenocarcinomas or high-grade dysplastic lesions possesses diagnostic utility in the evaluation of pancreatic cysts.

## Methods

### Cyst fluid samples

With the approval of the Stanford University Human Subjects Institutional Review Board, a pancreatic cyst fluid bio-repository has been maintained since July 2008. Patients evaluated at Stanford Hospital and Clinics for endoscopic ultrasound or surgery for pancreatic cysts were offered participation in the study. Cyst fluid was collected at the time of endoscopic ultrasound and/or surgery. Patients with a cyst large enough (typically greater than 1 cm) to provide cyst fluid beyond what was required for clinical evaluation was immediately placed on ice, aliquoted, and stored at -80°C. Clinical evaluation of the cyst fluid primarily involved 500 microliters of fluid for CEA analysis. Testing for amylase was left to the clinical discretion of the gastroenterologist or surgeon. When an intracystic nodule was seen, the nodule underwent fine needle aspiration for tissue diagnosis. All samples were aliquoted and frozen at -80°C within 30 minutes of collection. All samples assayed were subjected to no more than two freeze-thaw cycles, which does not affect the assay's reproducibility.

### Diagnosis of pancreatic cysts

Cyst diagnosis was determined by surgical pathology or cytology. In each of the surgically resected cases, histology slides were independently evaluated by a pathologist (RKP) for the histology type and grade of the neoplasm. All cases of IPMN and MCN were subclassified based on the grade of dysplasia: low-grade, intermediate-grade, and high-grade, using the WHO classification [17]. In this study, the definition of cancer included cystic lesions with high-grade dysplasia. Benign mucinous cysts included MCN or IPMN lesions with low- or intermediate-grade dysplasia.

### AREG ELISA

Researchers (M.T.T., A.W.L.) blinded to the patients' diagnoses conducted the AREG ELISAs. Cyst fluid AREG was determined using a two-antibody sandwich ELISA (DY262, R&D systems, Minneapolis, MN) according to the manufacturer's instructions. Standard curves were reproducible over a dynamic range of 5-2,000 pg/ml. Briefly, 100 microliter (ul) of sample was required for analysis and added to a 96-well ELISA plate (Fisher Scientific, Pittsburg, PA) that had been pre-coated with the capture antibody. After incubation with the detection antibody and streptavidin-HRP, the signal was developed by the addition of 3,3',5,5'-tetramethylbenzidine (TMB, Thermo Scientific, Rockford, IL), followed by the addition of a stop solution, and quantified by absorptive spectrophotometry at 450 and 562 nm on an automatic plate reader (Biotek, Winooski, VT). Assays for each sample were performed on serially diluted aliquots and performed in duplicate. The diluent consisted of 1% bovine serum albumin in phosphate buffered saline, pH 7.3. Dilutions within the assay's linear range on the standard curve were chosen. Data demonstrating that the ELISA specifically measures the AREG gene product was previously established [16].

### Statistical analysis

Comparisons between mucinous and non-mucinous cysts and benign mucinous and malignant mucinous cysts were performed. Based on a non-normal distribution of AREG levels by cyst type, the non-parametric Kruskal-Wallis test was used to compare AREG levels between the multiple categories of cysts. The Wilcoxon rank-sum test was used for comparison of 2 cyst types. A receiver operator curve was generated to characterize the accuracy of cyst fluid AREG to diagnose malignant mucinous cysts. When a significant difference was observed, a threshold with highest diagnostic accuracy was used to report the sensitivity and specificity of AREG. Statistical analysis was performed using STATA 11.0 (College Station, TX).

## Results

### Patients and cyst types

Thirty-three patients with pancreatic cysts were evaluated (Table 1). The mean age was 61 (range 33 - 83) and 54% (18 of 33) were males. The median cyst size was 2.8 cm (interquartile range [IQR] 2.0 - 4.4 cm). A histological diagnosis was conferred by surgical pathology for 30 samples and by cyst aspiration cytology for 3 samples. Among the 30 surgical pathology samples, there were 5 adenocarcinomas, 4 cysts with high-grade dysplasia (all MD-IPMN), 15 benign mucinous cysts (MCN = 3, BD-IPMN = 9, and MD-IPMN = 3), and 6 non-mucinous cysts (SCN = 4, PC = 1, squamous cyst = 1). Histological samples conferred only by cytology (n = 3) were cysts associated with unresectable adenocarcinoma.

**Table 1:** Summary of Patient and Cyst Characteristics

Total Patients	33
Median Age, years (range)	61 (33- 83)

Gender: Male/Female	18 (54%)/15 (46%)

Median Cyst Size, cm (IQR)	2.8 (2.0 - 4.4)

Non-Mucinous	6

SCN (n = 4)	

Pseudocyst (n = 1)	

Other (n = 1)	

Benign Mucinous	15

IPMN BD (n = 9)	

IPMN MD (n = 3)	

MCN (n = 3)	

Cancer (in situ)	12

High Grade (n = 4)	

Invasive (n = 8)	

### Diagnostic accuracy of AREG

**Figure 1:**
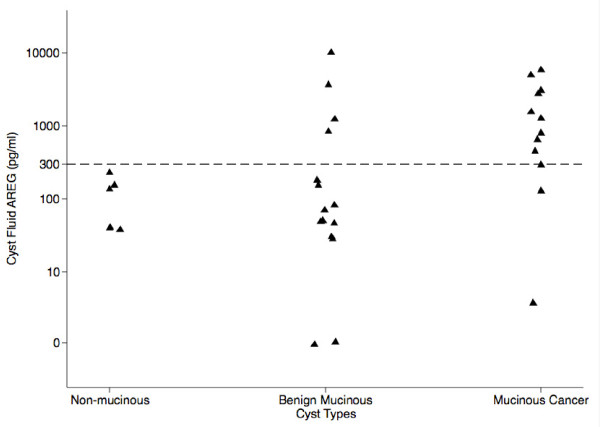
**Scatter plot of cyst AREG by non-mucinous, benign mucinous, and malignant mucinous cysts**.

Scatter plots of cyst AREG levels by cyst type are shown in Figure 1. The median (interquartile range, IQR) cyst AREG levels for non-mucinous cysts, benign mucinous cysts, and cancerous cysts were 85 pg/ml (47-168), 63 pg/ml (30-847), and 986 pg/ml (417-3160), respectively. Table 2 summarizes cyst AREG values by each type of cyst. No significant difference in AREG levels was appreciated between non-mucinous and mucinous cysts. When mucinous cysts were divided between benign and cancerous cysts a significant difference in cyst AREG levels was observed (*p *= 0.025).

**Table 2:** Summary of Cyst Fluid AREG performance by Cyst Types

Cyst Type (n = 33)	Median AREG (pg/ml)	IQR (pg/ml)
Non-Mucinous (n = 6)	85	47-168

SCN (n = 4)	48	44-109

Pseudocyst (n = 1)	227	

Other (n = 1)	121	

Benign Mucinous (n = 15)	63	30-847

IPMN BD (n = 9)	48	29-63

IPMN MD (n = 3)	847	71-9041

MCN (n = 3)	202	42-1030

Cancer (in situ)	986	417-3160

High Grade (n = 4)	417	214-546

Invasive (n = 8)	2047	986-4367

**Figure 2:**
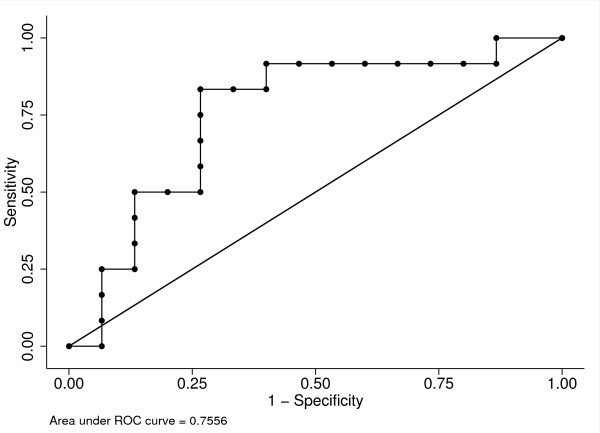
**ROC curve analysis of AREG to differentiate benign mucinous from malignant mucinous cysts**.

Based on the difference of cyst AREG levels between benign mucinous and mucinous cancers, a receiver operator curve (ROC) was generated to determine an optimal threshold to diagnose mucinous cancers (Figure 2). As a summary measure of diagnostic accuracy, the area under the ROC was 0.76 (95% CI 0.56-0.95). At an AREG threshold of greater than 300 pg/ml, the diagnostic accuracy for cancer was 78% with a sensitivity of 83% and specificity of 73%. With the prevalence of cancer of 32% in the sample, the positive and negative predictive value was 71% and 85%, respectively.

Further clinical details on the 12 patients with cancer in this sample are highlighted in Table 3. Four patients had high-grade dysplastic lesions, and included 3 MD-IPMN. The majority of patients (10 out of 12) had symptoms (i.e. jaundice, weight loss, abdominal pain) associated with cancer. The majority of patients had imaging evidence of a nodule within the cyst or an associated mass (8 out of 12). Two out of the 12 cases had *AREG *levels below 300 pg/ml. One case (*AREG *= 125) was an intraductal oncocytic papillary neoplasm and the other case (*AREG *= 4) was a 1.5 cm cyst adjacent to a pancreatic adenocarcinoma.

**Table 3:** Summary Table of 12 patients with histological diagnosis of Cancer (includes high grade dysplasia)

Patient Age/Gender	Symptomatic	Cyst Size (cm)	Location	Mural Nodule/Mass	AREG level (pg/ml)	CEA level (ng/ml)	Diagnosis
39/F	Yes	2.8	Tail	Yes	125	15	Intraductal Oncocytic Papillary Neoplasm

72/M	No	4.6	Body	No	303	2298	Main Duct IPMN with High-Grade Dysplasia

78/M	Yes	N/A	Diffuse	No	523	N/A	Main Duct IPMN with High-Grade Dysplasia

65/M	Yes	N/A	Diffuse	No	560	N/A	Main Duct IPMN with High-Grade Dysplasia

60/M	No	1.5	Body	Yes	4	1245	Adenocarcinoma

83/M	Yes	3.0	Body	Yes	694	42979	Adenocarcinoma

60/M	Yes	3.0	Tail	Yes	1279	N/A	Adenocarcinoma

60/F	Yes	3.2	Tail	Yes	1567	11962	Adenocarcinoma

66/F	Yes	2.6	Head	Yes	2527	N/A	Adenocarcinoma

70/M	Yes	N/A	Diffuse	No	3794	N/A	Colloid Carcinoma

51/M	Yes	3.0	Head	Yes	4940	N/A	Adenocarcinoma

65/M	Yes	7.5	Head	Yes	6458	2501	Adenocarcinoma

## Discussion

A biomarker that can accurately and reliably distinguish cancer or high-grade dysplasia among mucinous pancreatic cystic neoplasms remains an important clinical need. The most accepted cyst fluid biomarker currently is CEA, which is good at differentiating mucinous from non-mucinous cysts. CEA, however, is not reliable for differentiating cancer or high-grade dysplasia among pre-malignant mucinous cysts. As a result, current practice relies on clinical and radiographic data to help clinicians decide which cystic lesions warrant immediate surgery over observation [18]. While helpful, cases of unnecessary surgery or missed opportunities to resect cancer occur [19-21].

AREG's discovery as a potential cyst fluid biomarker arose from observations of increased Anterior Gradient 2 (*AGR2*) gene expression among pancreatic adenocarcinomas [13]. *AGR2 *is a highly conserved gene that is associated with mucus secreting cells. *AGR2 *stimulates adenocarcinoma cell growth and supports the development of many features associated with malignant transformation [14,15]. Closer examination of the gene expression studies showed that *AGR2 *expression was significantly higher in MCN cysts compared to SCA lesions. Recent studies revealed that *AREG*, a secreted epidermal growth factor receptor ligand, is specifically induced by AGR2 [16].

In this study, we examined the diagnostic utility of AREG in pancreatic cyst fluid and observed no difference in cyst AREG concentrations between non-mucinous and benign mucinous cysts. Malignant mucinous cysts that included high-grade dysplastic lesions, however, expressed a significantly higher AREG level (median 986 pg/ml) compared to benign mucinous cysts (median 63 pg/ml) and non-mucinous cysts (median 85 pg/ml). By receiver operator curve analysis, an AREG level of 300 pg/ml provided a diagnostic accuracy for cancer of 78% (sensitivity 83%, specificity 73%). The higher cyst AREG levels observed in malignant cysts is likely a function of the total cellular mass of AREG producing cells. As a benign cyst transitions to a malignant cyst, a hallmark of dysplasia includes a change from simple to stratified epithelium. We hypothesized that this results in a significant increase in the cellular mass of a cyst leading to increased cyst AREG expression. The similarities in cyst AREG levels between non-mucinous and benign mucinous cysts may be related to the physiologic expression of AREG as part of a reparative process in combination with a smaller cellular mass of mucin producing cells. Recent studies have determined that AREG serves an important role in tissue repair after damage in the gastrointestinal tract [22,23].

Because cyst CEA is fairly accurate in differentiating non-mucinous from mucinous cysts, the diagnostic utility of combining both CEA and AREG was considered. There were 21 of the 33 samples where cyst fluid CEA and AREG levels were available for analysis. The median (IQR) CEA levels for the 4 non-mucinous cysts, 11 benign mucinous, and 6 malignant mucinous cysts were 127 ng/ml (36-844), 1294 ng/ml (171-8600), and 2400 ng/ml (1245-11962), respectively. Mucinous cysts (n = 17) had an elevated CEA (median (IQR) 1311 ng/ml (277-8600)) compared to non-mucinous cysts (n = 4) (126 ng/ml (36-844) (*p *= 0.09). Although this difference was not statistically significant, this is likely due to the small sample size. Using a cutoff of 192 ng/ml, the sensitivity and specificity of CEA to differentiate non-mucinous from mucinous cysts was 76% and 75%, respectively- an observation similar to previous reports [7,24]. The small size of this sample may also explain why no difference in sensitivity and specificity for cancer was observed when combining AREG and CEA compared to AREG alone. When an AREG threshold of 300 pg/ml was used for the diagnosis of malignant mucinous cysts, the sensitivity was 67% and the specificity 80%. When AREG was sequentially tested only on pancreatic cysts with a CEA level greater than 192 ng/ml, neither the sensitivity nor specificity changed for cancer.

There are several features of this study that limit the generalizability of these observed results. First, this is a retrospective single tertiary center with a relatively small sample of cyst fluid samples. The small sample size is due in part to restricting the study to surgical patients. Although recruitment was difficult because patients with pancreatic cysts often do not undergo surgery, it was felt that as an initial proof-of-concept study, the use of pathology and surgically resected samples was a necessary gold standard to establish the correct diagnosis. As a result, the impact of a small sample size (in particular the limited cases of non-mucinous cysts) may include inadequate power to demonstrate a difference between non-mucinous and mucinous AREG levels should one truly exist. Second, the 12 cancer cases (including high grade dysplasia) were relatively advanced cases and could likely be identified by current practices without cyst AREG. It is unclear how AREG will perform in cases when imaging and clinical characteristics are non-specific. Many of these limitations, however, can be addressed in the future with prospective, longitudinal validation incorporating a larger sample size and multi-center collaboration.

## Conclusions

The present study represents the translation of recent discoveries in the basic biology of adenocarcinomas to clinical utility in the evaluation of pancreatic cysts. The study reports the discovery of AREG, a secreted epidermal growth factor receptor ligand, as a biomarker with potential diagnostic utility for diagnosing and managing pancreatic cystic neoplasms. Specifically, cyst AREG levels may help accurately identify those cysts with cancer and high-grade dysplastic lesions that require immediate surgical attention. Although not a serum-based test, EUS mediated acquisition of the 100 microliters of fluid necessary for analysis is within current practices for managing pancreatic cysts, and will facilitate validation in future studies.

## Competing interests

The authors declare that they have no competing interests.

## Authors' contributions

Study concept and design - MTT, AD, AG, WGP, JVD, and AWL; Acquisition of samples - RKP, SK, BCV, JAN, GAP, SB, JVD, AMC, SF, BAS, RV, and WGP; Analysis and interpretation of data - MTT, RKP, SK, AWL, WGP; Drafting of the manuscript - MTT, AWL, and WGP; Statistical analysis - WGP; Obtained funding - AWL; Co-senior authors and study supervision - AWL and WGP All authors read and approved the final manuscript.

## Pre-publication history

The pre-publication history for this paper can be accessed here:

http://www.biomedcentral.com/1471-230X/12/15/prepub
